# Lycopene mitigates T-2 toxin-induced systemic inflammation and oxidative stress in association with gut microbiota and SCFAs regulation in mice

**DOI:** 10.1007/s44154-026-00331-3

**Published:** 2026-09-10

**Authors:** Saber Y. Adam, Wael Ennab, Cuipeng Zhu, Long Yuan, Abdelkareem A. Ahmed, Mohamed Osman Abdalrahem Essa, Hosameldeen Mohamed Husien, Ahmed A. Saleh, In Ho Kim, Hao-Yu Liu, Demin Cai

**Affiliations:** 1 Guangling College, Yangzhou University, Yangzhou, 225009 People’s Republic of China; 2 College of Animal Science and Technology, Yangzhou University, Yangzhou, 225009 People’s Republic of China; 3 Biomedical Research Institute, Darfur University College, South Darfur State, Nyala, Sudan; 4 College of Veterinary Medicine, Yangzhou University, Yangzhou, 225009 Jiangsu China; 5 Animal and Fish Production Department, Faculty of Agriculture (Al-Shatby), Alexandria University, Alexandria City, 11865 Egypt; 6 Department of Animal Resource and Science, Dankook University, Cheonan-Si 31116, Choongnam, Republic of Korea; 7 Joint International Research Laboratory of Agricultural & Agri-Product Safety, The Ministry of Education of China, Yangzhou University, Yangzhou, 225009 People’s Republic of China; 8 Jiangsu Key Laboratory of Zoonosis, Yangzhou University, Yangzhou, 225009 People’s Republic of China; 9 Jiangsu Key Laboratory of Animal Genetic Breeding and Molecular Design, Yangzhou University, Yangzhou, 225009 People’s Republic of China

**Keywords:** BALB/c mice, Lycopene, T-2-trichothecene, Oxidative stress, Inflammation, Gut microbiota and SCFAs

## Abstract

T-2 toxin is a trichothecene mycotoxin produced by *Fusarium* species and can cause disease after contaminated food or feed ingestion. This study aimed to investigate the impact of lycopene (Ly) on systemic inflammation, oxidative stress, and dysbiosis in mice exposed to T-2 toxin. A total of 20 male BALB/c mice (6 weeks old, weighing 23.5 ± 0.32 g) randomly divided into four groups (*n* = 5): Control (Cont), Ly, T-2, and T-2 + Ly groups. Findings revealed that Ly enhanced the body weight, feedd, and water intake that have been reduced by T-2. Ly increased the villus height (VH) and VH: crypt depth (CD) and decreased CD and villus width (VW) in ileum compared to the T-2 group. In alpha diversity, *Staphylococcus* and *Corynebacterium* were the most prevalent in Cont group; *Staphylococcus*, and *Lactobacillus*, were the most prevalent in T-2 group; *Candidatus-Arthromitus* and *Staphylococcus* were the most prevalent in T-2 + Ly group. Moreover, T-2 mice had significantly (*p* < 0.05) greater levels of interleukin-1 beta (IL-1β), interferon gamma (IFN-γ), Interleukin-17 (IL-17), and tumor necrosis factor-alpha (TNF-α) than Cont, while the Ly significantly (*p* < 0.05) lowered their concentration. The T-2 mice had significantly (*p* < 0.05) higher levels of reactive oxygen species (ROS) and malondialdehyde (MDA), while their catalase (CAT),, glutathione (GSH), adenosine triphosphate (ATP), and superoxide dismutase 1(SOD1) activities were significantly (*p* < 0.05) lower than Cont. Ly significantly (*p* < 0.05) restored their concentration. Moreover, there was a significant reduction (*p* < 0.05) of hexanoic, butyric, acetic, pentanoic, and Heptanoic acids in the T-2 mice compared to Cont. In contrast, treatment with Ly was found to significantly restore their levels compared to T-2 group. We conclude that Ly administration in the context of T-2–induced toxicity may help attenuate systemic oxidative stress and inflammation, possibly through modulation of gut microbiota and fecal short-chain fatty acids (SCFAs).

## Introduction

Mycotoxins are secondary metabolites produced by filamentous fungi belonging to several genera, including *Aspergillus, Penicillium, Fusarium,* and *Alternaria* (Rasmussen et al. 2010; Gallo et al. 2021). These compounds are regarded as one of the major risk factors in agricultural products and have been the subject of investigation in a number of countries (Singh and Mehta 2020). The *Fusarium* genus produces T-2 toxin as a secondary metabolite, which is commonly present in cereals throughout many countries. Food or feed contaminated with trichothecene (T-2) toxin can harm both human and animal tissues and organs (Zhang et al. 2021). According to international guidelines, an individual's permissible daily intake of T-2 toxin cannot surpass 100 ng/kg body weight (BW) (Weidner et al. 2012). The 2018 Biomin Global Report states that among 8721 different types of agricultural products from 75 countries, the average concentration of T-2 toxin was 25 µg/kg, with a detection rate of up to 23% (Schmidt et al. 2018). T-2 toxin may remain in animal tissues, meat, eggs, and milk throughout the food chain, posing a threat to human health by raising the possibility of fatal alimentary toxic aleukia and Kaschin-Beck disease (Lei et al. 2016). There is mounting proof that T-2 toxin exposure can harm both humans and animals in a number of ways, including immunotoxicity (Wu et al. 2020), neurotoxicity (Dai et al. 2019), cardiotoxicity (Wang et al. 2023a), reproductive toxicity (Lee and Park 2023), and nephrotoxicity (Zhang et al. 2022). Consequently, in order to find efficient preventative and control methods, a thorough investigation of its harmful molecular pathways is required.

Inflammation and systemic oxidative stress are important related processes in a number of diseases and physiological states. While oxidative stress is caused by an imbalance between the body's antioxidant defense mechanisms and the production of reactive oxygen species (ROS), systemic inflammation involves the activation of immune responses throughout the body (Hertiš Petek et al. 2022). Many diseases can emerge and progress as a result of the interaction between these two processes, which can further worsen tissue damage (Liu et al. 2025). There are several ways in which oxidative stress can cause inflammation. ROS can activate inflammatory signaling pathways, such as NF-κB and NLRP3 inflammasomes, which can result in the production of pro-inflammatory cytokines (Kuntić et al. 2024). The inflammatory response is further intensified by these cytokines, which include tumor necrosis factor-alpha (TNF-α), interleukin-1β (IL-1β), and interleukin-6 (IL-6) (Ito et al. 2019). Because immune cells release ROS during the inflammatory response, inflammation can also increase oxidative stress (Dharshini et al. 2023). Chronic tissue damage may result from this self-reinforcing cycle of inflammation and oxidative stress. Several medical conditions, including cancer, metabolic syndrome, neurodegenerative diseases, cardiovascular diseases, and osteoporosis, have been linked to these processes (Shang et al. 2023; Balan et al. 2024; Liu et al. 2025).

The mammalian gastrointestinal tract hosts to a diverse range of bacteria, archaea, fungi, viruses, and other microorganisms known as the gut microbiota. This microbial community's composition varies depending on the host, changes over the course of a person's lifespan, and can be altered by both exogenous and endogenous factors (Sekirov et al. 2010). *Firmicutes, Bacteroidota, Actinobacteria,* and *Proteobacteria* represent the majority of the gut microbiota, with *Firmicutes* and *Bacteroidota* accounting for over 90%*.* These microorganisms are essential for the metabolism and digestion of food, the preservation of the intestinal barrier, and immune system regulation. Approximately 100 trillion (10^14^) bacteria reside in the gut, and their gene pool is greater than that of the human genome (Zhang et al. 2024). Different physiological conditions make it difficult to define what constitutes a "healthy" gut microbiota, even though a healthy microbiome is usually diversified and functionally stable (Fan and Pedersen 2021; Yang et al. 2023). Maintaining host health relies on the gut microbiota, which supports digestion, strengthens the intestinal epithelial barrier, and prevents pathogen invasion (Zhao et al. 2023), and improving immunity, and tissue development (Fan and Pedersen 2021). The bacteria in the gut can synthesize some essential and non-essential amino acids, create a wide range of vitamins, and biotransform bile (Vyas and Ranganathan 2012). Additionally, metabolites of the gut microbiota can alter host physiology and metabolism, making it easier to digest food and produce energy from indigestible substrates (Pessôa et al. 2023). For instance, indigestible fiber can be used to produce short-chain fatty acids (SCFAs) (Shreiner et al. 2015; Rinninella et al. 2019). The intestinal mucosa is fueled by SCFAs, which also preserve homeostasis and control immunological responses to stop inflammation and cancer (Wells et al. 2011; Smith et al. 2013). With their systemic effects, SCFAs can affect oxidative stress in the brain, kidneys, liver, and other organs (He et al. 2024).

Natural compounds have gained attention in reducing mycotoxin-related diseases (Li et al. 2024). Lycopene (Ly) is a bright red carotenoid hydrocarbon, present in tomatoes as well as other red fruits and vegetables such as papaya, watermelons, red carrots, and grapefruits (Róvero Costa et al. 2019). Ly is widely known for its anti-inflammatory and antioxidant properties, and it can affect important bodily metabolic processes (Han et al. 2016). In this context, it was recently discovered by Al-Brakati et al. (Al-Brakati et al. 2021) that the production of selenium nanoparticles utilizing Ly had renoprotective effects on acute kidney injury via antioxidant, anti-inflammatory, anti-necroptotic, and anti-apoptotic properties. Moreover, Ly protects the biomolecules of the potentially fatal cells such as DNA, lipids, and lipoproteins from oxidation (Anlar and Bacanli 2020; Bin-Jumah et al. 2022). However, no prior studies have examined Ly's protective role against T-2-induced systemic inflammation and oxidative stress and link gut microbiota dynamics to functional outcomes via SCFAs in mice. This work therefore aimed to determine if Ly may reduce systemic inflammation and oxidative stress in mice caused by T-2 toxin and to examine the underlying mechanisms, with a particular emphasis on the modulation of gut microbiota and fecal SCFAs.

## Results

### Body weight, feed, and water intake

**Fig. 1:**
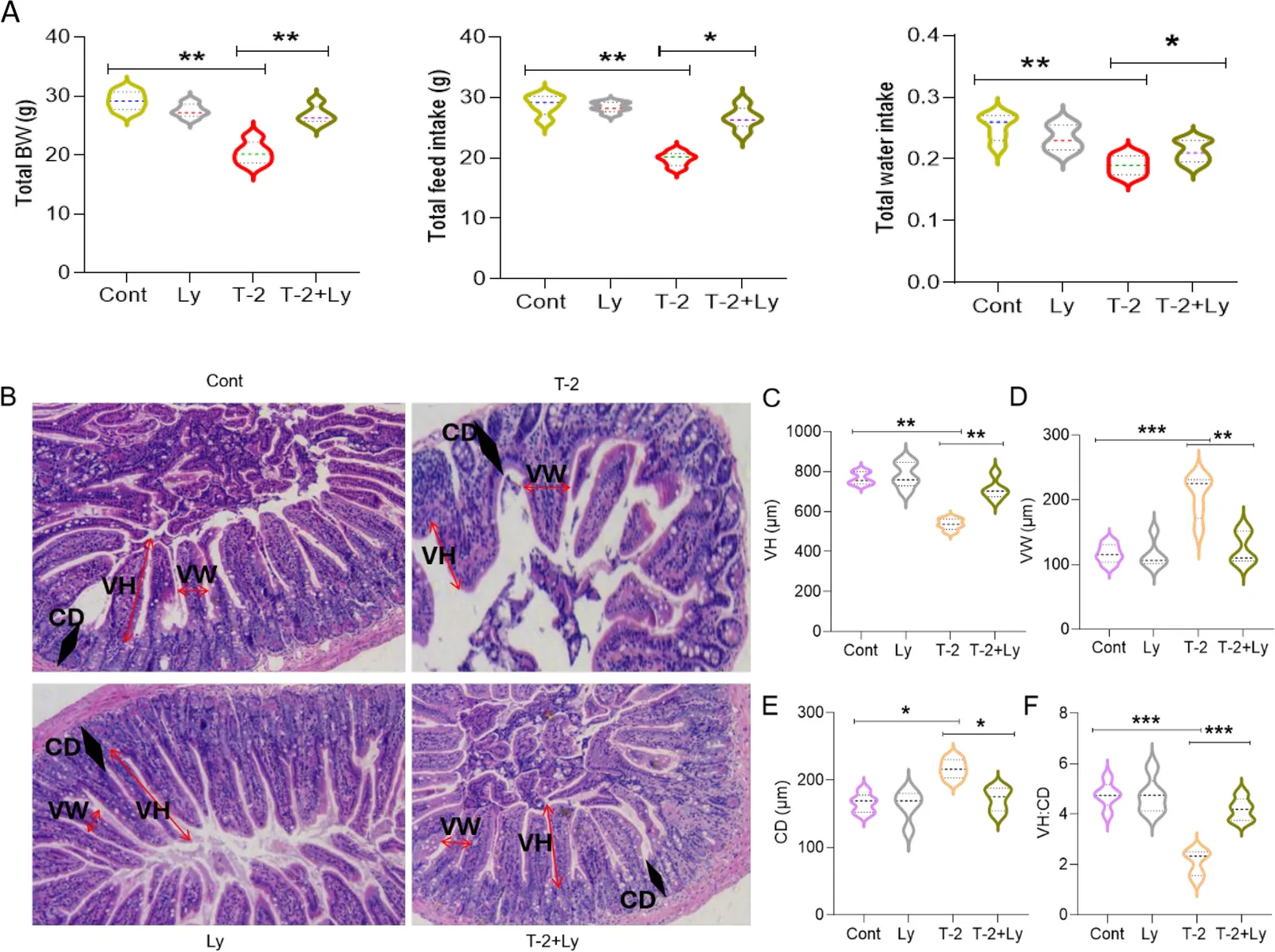
Shows the effects of Ly supplementation in mice exposed to T-2 toxin. The body weight, food, and water consumption (**A**); Histopathology (H&E stain) of the ileum with magnification (20 ×) and scale bar (100 µm) (**B**); Villus height (**C**) (VH); Villus width (VW) (**D**); Crypt depth (CD) (**E**); Villus height/Crypt depth (VH/CD) (**F**). Data were presented as means ± SD. * *p* < 0.05, ** *p* < 0.01, *** *p* < 0.001. *n* = 5

The (Fig. 1A) illustrates how the experimental mice's body weight, feed, and water intake were affected by T-2 toxicity. The total mice's body weight, feed, and water intake were lower in the T-2 group than the Cont group (*p* < 0.05). However, a significant (*p* < 0.05) increase in body weight, feed and water consumption was found when comparing the T-2 + Ly group to the T-2 group.

When compared to the Cont group, the T-2 group's VH and VH: CD were significantly lower (*p* < 0.05), yet when Ly was administered to mice, their VH and VH: CD were significantly higher (*p* < 0.05) than those of the T-2 group (Fig. 1C, F). Furthermore, compared to the Cont group, the T-2 group's CD and VW of the ileum were substantially higher (*p* < 0.05), and the beneficial effects of Ly supplementation were seen in comparison to the T-2 group (Fig. 1D, E).

### Gut microbial diversity analysis

**Fig. 2:**
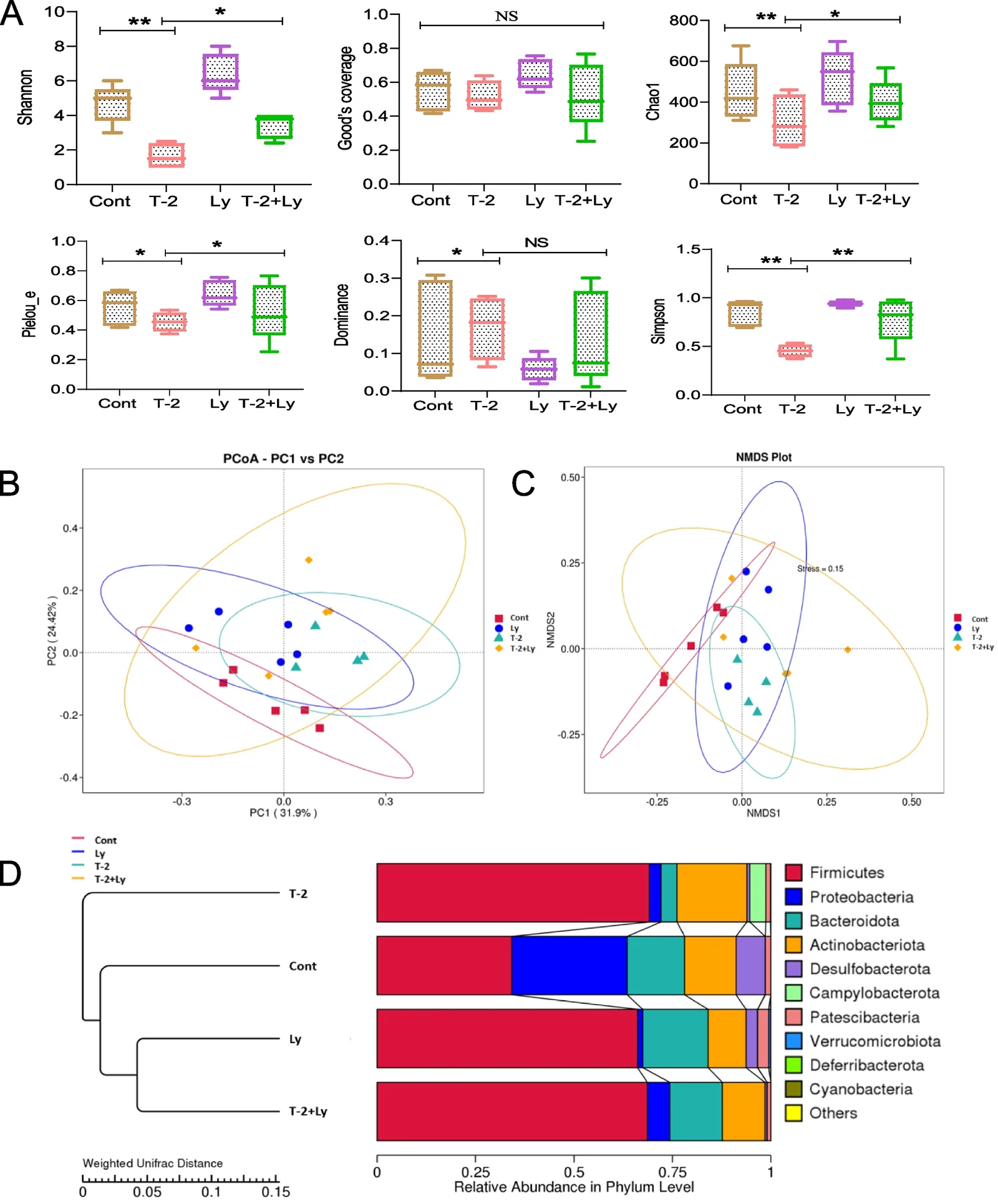
Shows the Shannon, Good’s coverage, Chao1, Pielou-e, Dominance, and Simpson of alpha diversity (**A**); PCoA analysis (**B**); NMDS plot analysis (**C**); and UPGMA plot (**D**) of the mice exposed to T-2 toxin and treated with Ly. Data were presented as means ± SD. * *p* < 0.05, ** *p* < 0.01, *** *p* < 0.001. *n* = 5

The alpha-diversity of the gut microbiota was assessed using multiple indices. The T-2 group showed significantly (*p* < 0.05) lower levels of Shannon, Chao1, Pielou-e, dominance, and Simpson indices compared to the Cont group. Following lycopene administration, the T-2 + Ly group exhibited significantly (*p* < 0.05) higher levels of these indices compared to the T-2 group, indicating that lycopene partially restored alpha-diversity metrics altered by T-2 exposure. Notably, Goods coverage showed no significant differences (*p* > 0.05) among the experimental groups (Fig. 2A).

Beta-diversity was assessed using multiple methods. PCoA based on Jaccard distance revealed distinct separation between the Cont and T-2 groups (Fig. 2B). NMDS analysis was performed, and the stress value was (0.15), indicating a good fit of the ordination (Fig. 2C). UPGMA clustering showed that samples with shorter branch lengths had higher similarity (Fig. 2D). Statistical comparisons of beta-diversity revealed that inter-group differences were significantly greater than intra-group differences (*p* < 0.05), as confirmed by ANOSIM (R = 0.7, *p* = 0.007), MRPP (A = 0.15908, *p* = 0.006), and ADONIS (R^2^ = 0.3572, F = 3.88992, *p* = 0.012) Table 1.

**Table 1:** Analysis of differences between groups in ANOSIM, MRPP, and ADONIS groups

A							
Groups				R-value		P-value	
Cont-Ly				0.604		0.012	
Cont-T + 2				0.7		0.007	
Cont-T2 + Ly				0.628		0.008	
Ly-T-2				0.68125		0.014	
Ly-T-2 + Ly				0.596		0.009	
T-2-T-2 + Ly				0.52188		0.017	
B							
Group		A	observed-delta		expected-delta	Significance	
Cont-Ly		0.12347	0.6327		0.72183	0.011	
Cont-T + 2		0.15908	0.53449		0.6356	0.006	
Cont-T-2 + Ly		0.15601	0.59204		0.70148	0.01	
Ly-T-2		0.13705	0.63876		0.74021	0.006	
Ly-T-2 + Ly		0.09725	0.68589		0.75978	0.02	
T2-T-2 + Ly		0.14711	0.59359		0.69597	0.013	
C							
Group		Df	SumsOfSqs	MeanSqs	F.Model	R2	Pr (> F)
Cont-Ly		1 (8)	0.75558 (1.68165)	0.75558 (0.21021)	3.59446	0.31002 (0.68998)	0.01
Cont-T + 2		1 (7)	0.61184 (1.10102)	0.61184 (0.15729)	3.88992	0.3572 (0.6428)	0.012
Cont-T-2 + Ly		1 (8)	0.82024 (1.528)	0.82024 (0.191)	4.29447	0.3493 (0.6507)	0.008
Ly-T-2		1 (7)	0.74356 (1.54571)	0.74356 (0.22082)	3.36731	0.3248 (0.6752)	0.012
Ly-T-2 + Ly		1 (8)	0.70771(1.97269)	0.70771(0.24659)	2.87003	0.26403 (0.73597)	0.007
T2-T-2 + Ly		1 (7)	0.66539 (1.39206)	0.66539 (0.19887)	3.34594	0.32341(0.67659)	0.022

**A** ANOSIM**:** The difference between groups was larger than the difference within the group, as indicated by the R-value, which was more than 0 and fell between −1 and 1. The reliability of the statistical analysis is reflected by the P-value, and *P* < 0.05 indicates that the statistics are significant. **B** MRPP**:** A large inter-group variation is indicated by a bigger Expect delta value, while a smaller Observe Delta value denotes a modest intra-group difference. When the difference between groups is more than the difference within the group, the value is greater than zero; conversely, when the value is less than zero, the difference within the group is bigger than the difference between groups. A significant difference is indicated by a significance value of less than 0.05. **C** ADONIS**:** Using the permutation test, this approach determines the statistical significance of the grouping and assesses the explanatory power of various grouping factors for sample differences

### Gut microbial composition analysis

**Fig. 3:**
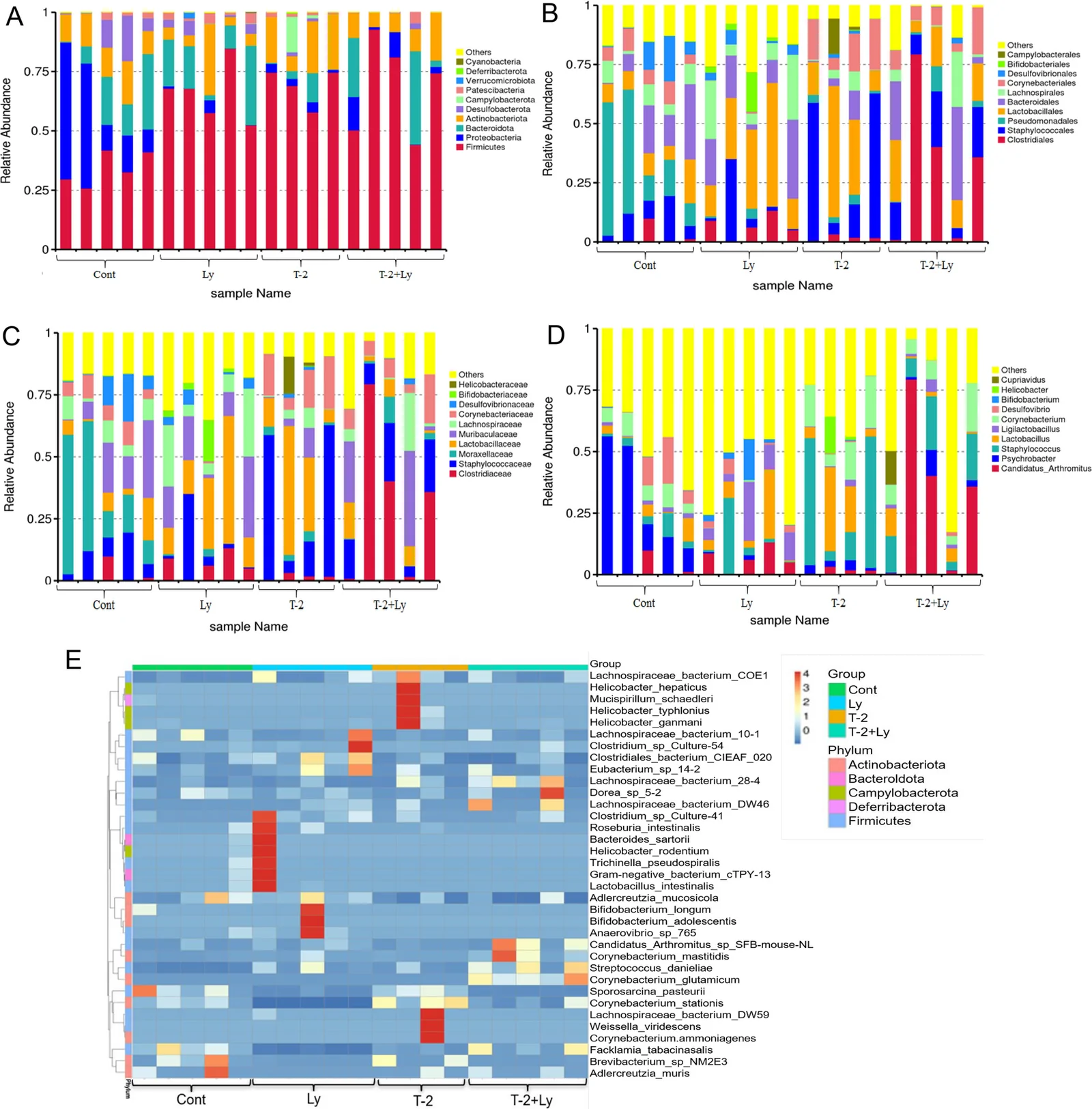
Demonstrates how Ly protects mice against imbalances in relative microbial abundance brought on by T-2 toxin gavage. Phylum-level relative abundance of fecal bacteria (**A**), order-level relative abundance of fecal bacteria (**B**), family-level relative abundance of fecal bacteria (**C**), genus-level relative abundance of fecal bacteria (**D**), and the clustering analysis-generated heat map also demonstrated the relative richness of these microbes (**E**). *n* = 5

Analysis of gut microbial composition revealed biologically meaningful shifts across experimental groups (Fig. 3A). At the phylum level, *Firmicutes* increased from 34.43% in the Cont group to 46.35% (Ly), 48.55% (T-2), and 49.11% (T-2 + Ly), while *Bacteroidota* decreased from 22.34% (Cont) to 20.18% (Ly), 18.11% (T-2), and 15.23% (T-2 + Ly). *Actinobacteriota* also declined, particularly in the Ly (11.43%) and T-2 + Ly (11.03%) groups compared to controls (16.23%).

At the order level (Fig. 3B), *Clostridiales* abundance shifted from 4.05% in controls to 9.25% (Ly), 4.19% (T-2), and 21.56% (T-2 + Ly), indicating a marked increase only in the combined treatment group. *Staphylococcales* remained relatively stable across groups (15.6% in Cont, 11.34% in Ly, 22.72% in T-2, 16.45% in T-2 + Ly), whereas *Lactobacillales* increased substantially in Ly (25.12%) and T-2 (24.34%) compared to controls (11.17%) before declining to 18.09% in T-2 + Ly.

Family-level composition (Fig. 3C) further distinguished the groups. The control group was characterized by *Staphylococcaceae* (15.67%), *Moraxellaceae* (28.92%), and *Lactobacillaceae* (10.23%). The Ly group showed enrichment of *Clostridiaceae* (9.47%), *Lactobacillaceae* (29.08%), and *Lachnospiraceae* (12.87%). In the T-2 group, *Staphylococcaceae* (22.46%), *Lactobacillaceae* (20.14%), and *Corynebacteriaceae* (12.97%) predominated. The T-2 + Ly group was distinctly marked by a large increase in *Clostridiaceae* (36.37%), along with *Staphylococcaceae* (14.09%) and *Muribaculaceae* (9.23%).

At the genus level (Fig. 3D), control mice were dominated by *Psychrobacter* (30.02%), Staphylococcus (9.15%), and *Corynebacterium* (13.03%). The Ly group showed high prevalence of *Candidatus-Arthromitus* (11.45%), *Ligilactobacillus* (14.85%), and Lactobacillus (13.54%). The T-2 group was characterized by Staphylococcus (35.06%), Lactobacillus (14.78%), and *Corynebacterium* (18.64%). In the T-2 + Ly group, *Candidatus-Arthromitus* (33.58%), Staphylococcus (21.47%), and *Corynebacterium* (17.06%) were most abundant. A heatmap created by clustering analysis confirmed these compositional differences (Fig. 3E). Future studies could investigate the functional roles of these taxa in gut health.

### LEfSe, correlation network, and t-test analysis

**Fig. 4:**
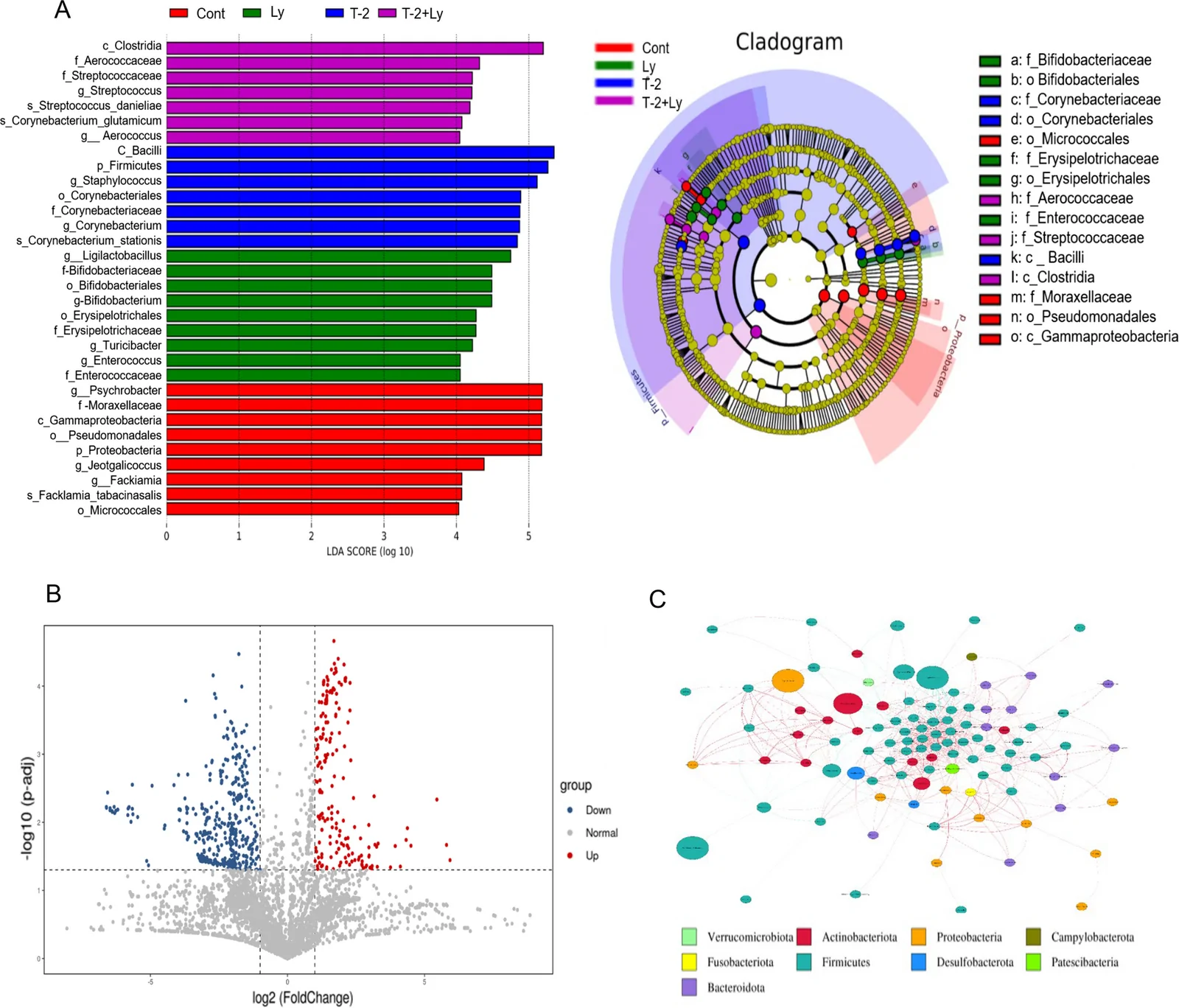
Shows the microbial abundances of mice feces exposed to T-2 toxin and treated with Ly, The statistical results of LEfSe include two parts, which are the LDA value distribution histogram and the phylogenetic branch plot (phylogenetic distribution) (**A**); Vvolcano map (**B**); Ccorrelation Aanalysis (Nnetwork Ddiagram) (**C**). *n* = 5

To comprehensively detect taxonomic composition differences across groups, we performed LEfSe analysis (including the histogram of LDA value distribution and the evolutionary branch diagram) at the family, order, genus, and species levels, in conjunction with cladogram scores (Fig. 4A). The analysis revealed distinct taxon enrichments that are biologically meaningful for understanding gut microbiome shifts under each treatment. In the control (Cont) group, *Psychrobacter, Moraxellaceae, Gammaproteobacteria, Pseudomonadales,* and *Proteobacteria* were predominant. The Ly group was characterized by significant enrichment of *Ligilactobacillus, Bifidobacteriaceae, Bifidobacteriales,* and *Bifidobacterium*, taxa commonly associated with beneficial gut effects. In contrast, the T-2 group showed predominance of *Bacilli*, *Firmicutes*, *Staphylococcus*, and *Corynebacteriales*. Finally, the T-2 + Ly group was distinguished by high abundance of *Clostridia*, *Aerococcaceae, Streptococcaceae,* and *Corynebacterium glutamicum*. Volcano plot of differentially expressed of 16S rRNA sequencing data (T-2 + Ly vs. T-2), each point represents an individual feature. The x-axis shows the log₂ (fold change), and the y-axis shows the -log₁₀(p-value). Features with log₂ (fold change) ≥ 1 and -log₁₀ (p value) ≥ 1.3 (equivalent to *p* < 0.05) were considered significantly upregulated (red, log₂ FC ≥ 1) or downregulated (blue, log₂ FC ≤ −1). Features not meeting these thresholds are shown in gray (Fig. 4B).

To explore potential co-occurrence patterns among bacterial phyla, we constructed a correlation network based on Spearman correlations of relative abundances (Fig. 4C). Edges were retained only when Spearman's rho > 0.6 and the FDR-adjusted *p*-value < 0.05. This analysis is biologically informative because it reveals which phyla tend to co-vary across samples, potentially indicating synergistic or shared ecological niches. Specifically, *Firmicutes* showed strong positive associations with *Proteobacteria* (rho = 0.94), *Actinobacteriota* (0.91), *Bacteroidota* (0.96), and *Patescibacteria* (0.81). *Actinobacteriota* was positively correlated with *Firmicutes* (0.93) and *Proteobacteria* (0.81). *Bacteroidota* was also positively associated with *Proteobacteria* (0.83) and *Firmicutes* (0.96). These high-magnitude positive correlations suggest that these phyla may respond similarly to experimental conditions or share functional dependencies within the gut environment.

**Fig. 5:**
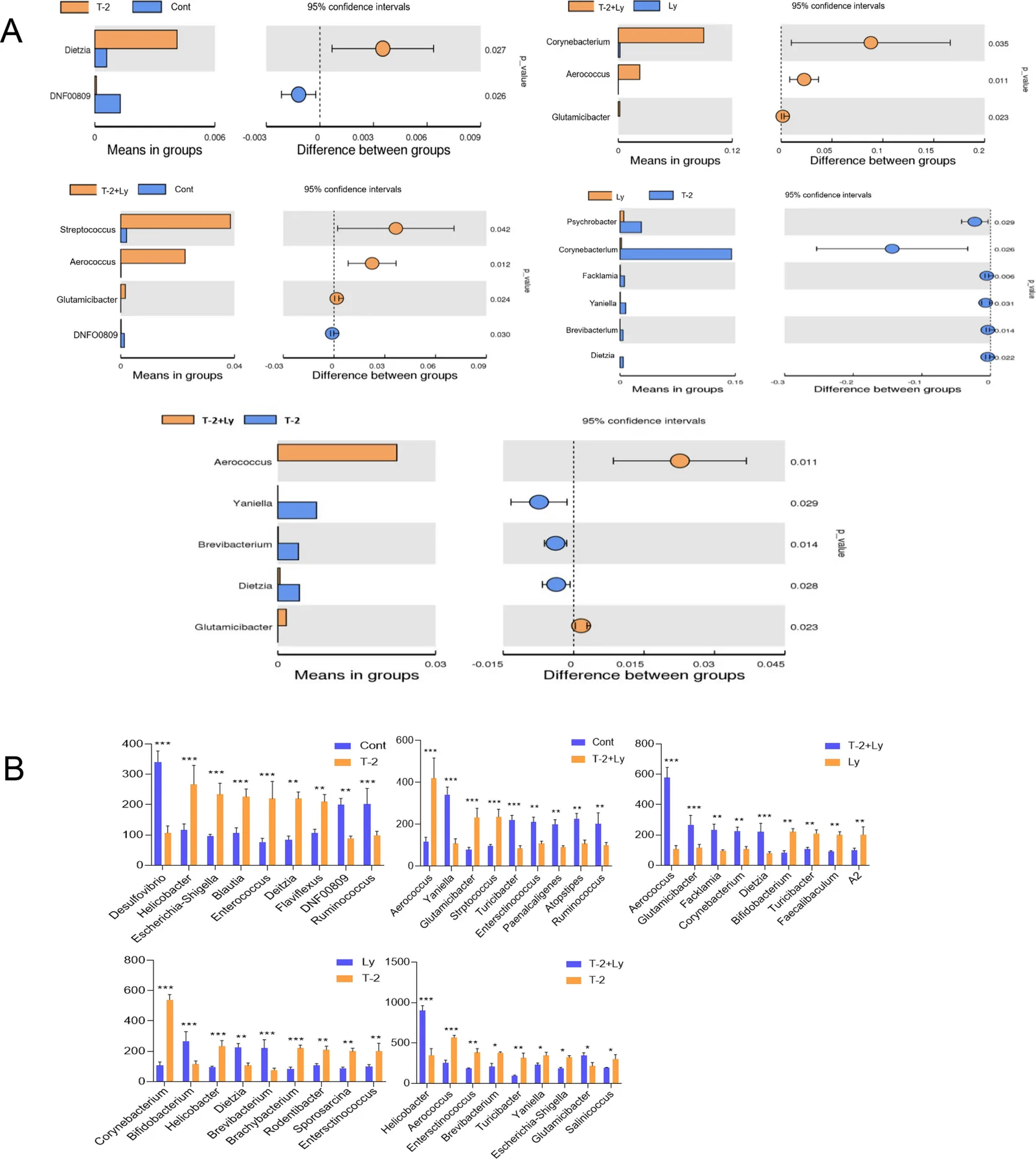
Shows the t-test results between the experiment groups, The figure on the left shows the abundance of species with significant differences between groups, and each bar in the figure represents the mean value of species in each group with significant differences in abundance between groups. The leftmost endpoint of each circle in the figure represents the lower limit of the 95% confidence interval for the mean difference, and the rightmost endpoint of the circle represents the upper limit of the 95% confidence interval for the mean difference. The center of the circle represents the difference of the mean, and the color of the circle represents the difference between groups of the corresponding species, (**A**). The MetagenomeSeq method was used to hypothesis test the species abundance data between groups and the results of the genus level are displayed, (**B**). *n* = 5

As showed in (Fig. 5A), the T-2 group displayed a significant (*p* < 0.05) decrease in DNF00809 and an increase in *Dietzia* in comparison to the Cont group. Comparing the fecal samples of the T-2 + Ly group to those of the Ly group, it was discovered that the relative abundance of *Corynebaterium*, *Aerococcus*, and *Glutamicibacter* had dramatically risen (*p* < 0.05). Furthermore, the T-2 + Ly group exhibited a higher relative abundance of *Corynebaterium*, *Aerococcus*, and *Glutamicibacter* compared to the Cont group (*p* < 0.05). The T-2 group's feces had significantly greater levels of *Psychrobacter* and *Corynebaterium* (*p* < 0.05) than the Ly group. *Aerococcus* and *Glutamicibacter* relative abundances were significantly (*p* < 0.05) lower in the T-2 group than in the T-2 + Ly group. In contrast, the relative abundances of *Yaniella*, *Brevibacterium*, and *Dietzia* were significantly (*p* < 0.05) greater in the T-2 group than in the T-2 + Ly group.

The MetagenomeSeq method was used to hypotheses test the species abundance data between groups in order to determine the p-value, which was then utilized to identify species that varied significantly between groups. The species that differed significantly between groups were then filtered based on the p-value, and the abundance distribution box plot of the various species between groups was created. By default, the genus level results are shown in (Fig. 5B). The amount of *Desulfovibrio, DNF00809* and *Ruminococcus* were decreased significantly (*p* < 0.05) in T-2 compared to Cont group, while the *Helicobacter, Escherichia-Shigella, Blautia, Enterococcus, Dietzia and Flaviflexus* were increased significantly (*p* < 0.05) in the T-2 group compared to the Cont group. Moreover, in contrast to the Cont group, the relative abundance of *Aerococcus, Glutamicibacter, and Spreptococcus* in the T-2 + Ly group was shown to be increased (*p* < 0.05), while the *Yaniella, Turicibacter, Entersctinococcus, Paenalcaligenes, Atopstipes and Ruminococcus* were deceased (*p* < 0.05) in T-2 + Ly compared to Cont. It was found that the relative abundance of *Aerococcus, Glutamicibacter, Facklamia, Corynebaterium,* and *Dietzia* in the fecal sample of the T-2 + Ly groups had increased significantly (*p* < 0.05) as compared with the Ly group, while the *Bifidobacterium, Turicibacter, Feacalibaculum,* and A2 in the fecal sample of the T-2 + Ly groups had decreased significantly (*p* < 0.05) compared to Ly group. When compared to the Ly group, the relative abundance of *Corynebaterium, Helicobacter, Brachybacterium, Rodentibacter, Sporosarcina,* and *Entersctinococcus* in the T-2 group was significantly increased (*p* < 0.05), in contrast, the *Bifidobacterium, Dietzia,* and *Brevibacteruim* relative abundance were significantly (*p* < 0.05) increased in Ly compared to T-2 group. Finally, the amount of *Helicobacter and Glutamicibacter* were significantly (*p* < 0.05) increased, while *Aerococcus, Entersctinococcus, Brevibacteruim, Turicibacter, Yaniella, Escherichia-Shigella,* and *Salinicoccus* were significantly (*p* < 0.05) decreased in T-2 + Ly compared to T-2 mice.

### Plasma pro-inflammatory cytokines

**Fig. 6:**
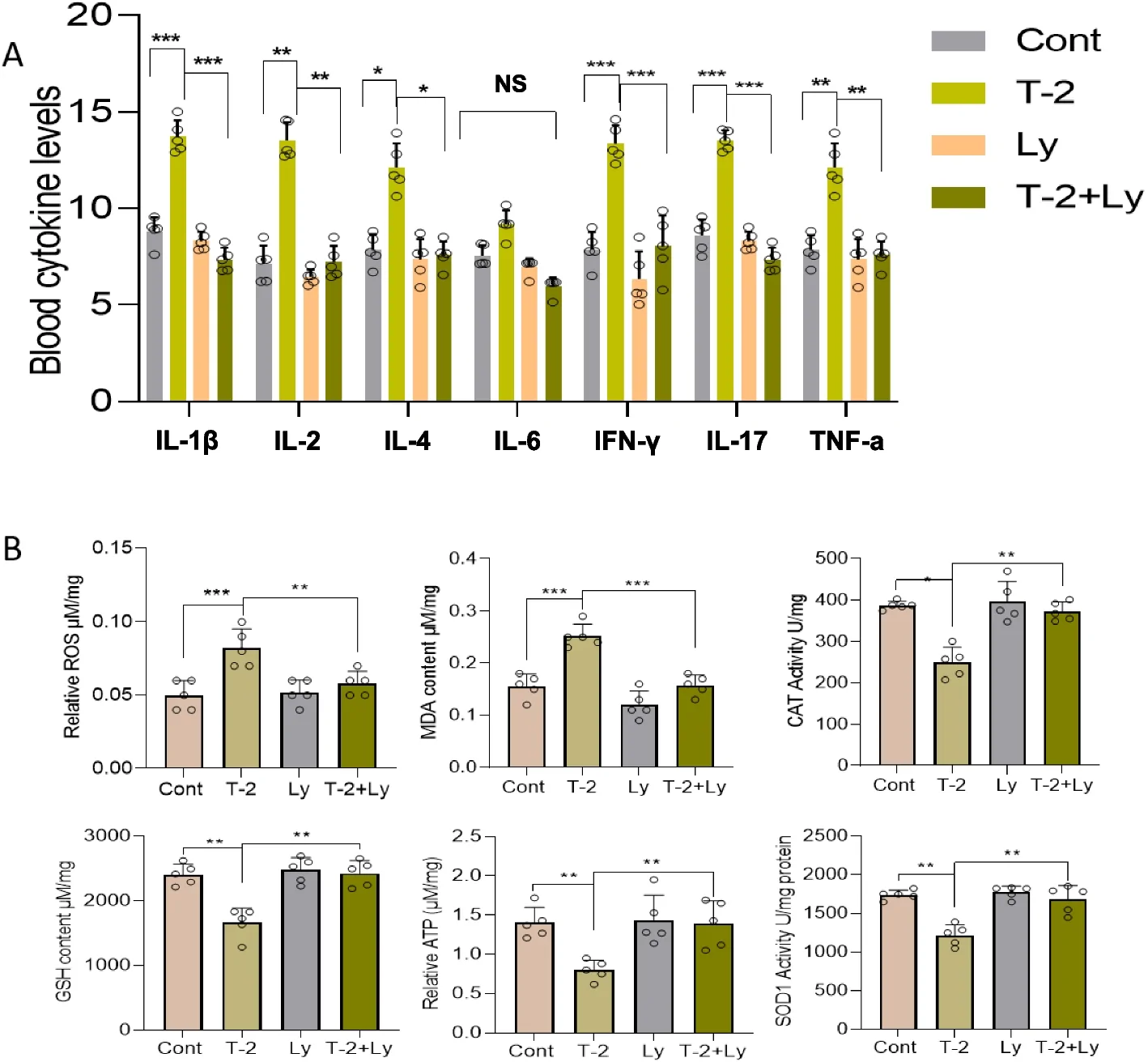
Effects of Ly on plasma inflammatory factors and oxidative stress indices in T-2 mice. (**A**) IL-1β, IL-2 and IL-4, IFN-γ, IL-17, and TNF-α. (**B**) ROS, MDA, CAT, GSH, ATP, and SOD1. Data were expressed as mean ± SD. * *p* < 0.05, ** *p* < 0.01, *** *p* < 0.001. *n* = 5

As shown in Fig. 6A, T-2 mice had significantly (*p* < 0.05) higher levels of IL-1β, IL-2, IL-4, IFN-γ, IL-17, and TNF-α compared to Cont mice. When compared to T-2 mice, the concentrations of these same cytokines (IL-1β, IL-2, IL-4, IFN-γ, IL-17, and TNF-α) were significantly (*p* < 0.05) reduced in T-2 + Ly mice. In contrast, IL-6 levels showed no statistically significant difference across any of the experimental groups (*p* > 0.05). Therefore, all measured cytokines except IL-6 were significantly altered by T-2 exposure and significantly improved (reduced) by Ly supplementation. These findings indicate that Ly may play an important role in reducing systemic inflammation in mice exposed to T-2 poisoning, although the effect does not extend to IL-6.

### Oxidative and antioxidant indexes

As illustrates in (Fig. 6B), the plasma ROS and MDA levels of the T-2 mice were significantly greater (*p* < 0.05) than those of the Cont group. CAT, GSH, ATP, and SOD1 activities, on the other hand, were significantly (*p* < 0.05) lower in the T-2 mice than in the Cont group. But in the plasma of the T-2 + Ly group compared to the T-2 group, Ly treatment significantly (*p* < 0.05) raised the levels of CAT, GSH, ATP, and SOD1 while decreasing the levels of ROS and MDA. This suggested that Ly supplementation may be essential for reducing systemic oxidative stress in mice exposed to mycotoxin poisoning.

### Short-chain fatty acid

**Fig. 7:**
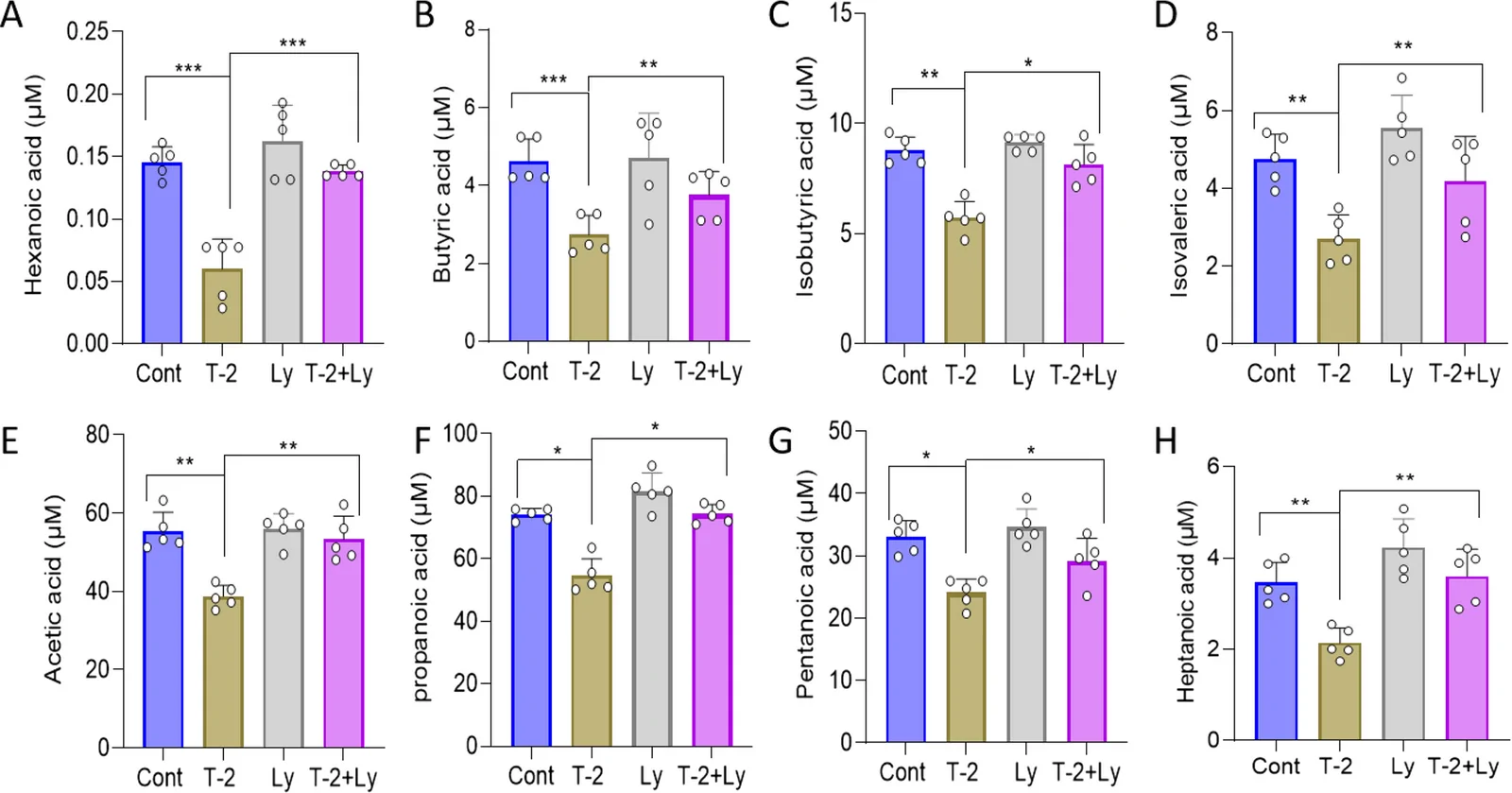
Shows the amount of short-chain fatty acids (SCFAs) in feces of mice exposed to T-2 toxin and treated with Ly, Levels of different types of SCFAs are reflected, such as (**A**) hexanoic; (**B**) butyric; (**C**) isobutyric; (**D**) isovaleric; (**E**) acetic acid; (**F**) propionic; (**G**) pentanoic; and (**H**) heptanoic acid. Data were presented as means ± SD. * *p* < 0.05, ** *p* < 0.01, *** *p* < 0.001. *n* = 5

As shows in (Fig. 7) that the concentrations of hexanoic acid, butyric acid, isobutyric acid, isovaleric acid, acetic acid, propionic acid, pentanoic acid, and heptanoic acid were significantly lower (*p* < 0.05) in the feces of the mice treated with T-2 than in the Cont group. The levels of hexanoic acid, butyric acid, isobutyric acid, isovaleric acid, acetic acid, propionic acid, pentanoic acid, and heptanoic acid were significantly restored in T-2 + Ly animals after treatment with Ly, in contrast to the T-2 group (Fig. 7A-H). This implies that by lessening the negative effects of T-2 toxin on gut bacterial activity or metabolism, Ly treatment can assist in restoring the SCFA levels to more normal or regulated levels.

### Correlation between oxidative stress, gut microbiota and SCFAs

To integrate our findings, we performed a correlation analysis to elucidate the interrelationships between systemic oxidative stress, gut microbiota composition, and fecal SCFA levels. Given the limited sample size (*n* = 20) and the number of pairwise comparisons, we applied False Discovery Rate (FDR) correction to control for the risk of false positives. Following FDR correction, several associations remained significant at a corrected threshold of q < 0.05, as presented in Table 2. These results should be interpreted as exploratory and hypothesis-generating. Notably, the oxidative stress marker ROS exhibited a strong positive correlation with the genus Helicobacter (*r* = 0.72, q < 0.001) and a negative correlation with the SCFA hexanoic acid (*r* = −0.69, q < 0.01). Conversely, the antioxidant enzyme SOD1 was positively correlated with several SCFAs, including butyric acid (*r* = 0.65, q < 0.01) and acetic acid (*r *= 0.61, q < 0.01), while showing a significant negative correlation with Helicobacter (*r* = −0.67, q < 0.01). Regarding microbial taxa, Bifidobacterium demonstrated a positive correlation with the antioxidant markers CAT (*r* = 0.58, q < 0.05) and SOD1 (*r* = 0.55, q < 0.05), and with key SCFAs like butyric acid (*r* = 0.63, q < 0.01). In contrast, Staphylococcus was negatively associated with acetic acid (*r* = −0.59, q < 0.01) and propionic acid (*r* = −0.56, q < 0.05). Furthermore, the SCFA butyric acid was negatively correlated with MDA levels (*r* = −0.60, q < 0.01), a marker of lipid peroxidation.

**Table 2:** Correlations between oxidative stress markers, gut microbiota, and short-chain fatty acids (SCFAs)

Parameter 1	Parameter 2	Correlation Coefficient (r)	*q* -value
**Oxidative Stress**	**Microbiota**		
ROS	*Helicobacter*	0.72	< 0.001
SOD1	*Helicobacter*	−0.67	< 0.01
CAT	*Bifidobacterium*	0.58	< 0.05
SOD1	*Bifidobacterium*	0.55	< 0.05
**Oxidative Stress**	**SCFAs**		
ROS	Hexanoic acid	−0.69	< 0.01
SOD1	Butyric acid	0.65	< 0.01
SOD1	Acetic acid	0.61	< 0.01
MDA	Butyric acid	−0.6	< 0.01
**Microbiota**	**SCFAs**		
*Bifidobacterium*	Butyric acid	0.63	< 0.01
*Staphylococcus*	Acetic acid	−0.59	< 0.01
*Staphylococcus*	Propionic acid	−0.56	< 0.05

*ROS* Reactive oxygen species, *MDA* Malondialdehyde, *CAT* Catalase, *SOD1* Superoxide dismutase 1

## Discussion

T-2 toxin is an important issue for food and feed safety and environment. Based on the previous research, T-2 does not affect cellular protein translation; rather, it inhibits the protein translation of mitochondria prior to mitochondrial membrane disintegration (Bin-Umer et al. 2011). T-2 toxicity has been shown to be largely caused by the activation of ROS generation and oxidative stress, which interferes with the function or synthesis of antioxidant enzymes (Wu et al. 2014). The gap in this research is the limited understanding of how lycopene mitigates T-2 toxin-induced oxidative stress through modulation of gut microbiota and fecal SCFAs in mice. However, the aim of this study is to investigate the protective effect of Ly intake and its regulation of oxidative stress via modulation intestinal microbiota and SCFAs during T-2 caused toxicity in mice.

Body weight (BW) (Zhang et al. 2017), feed, and water consumption are the basic signs of health; weight loss, reduced feed and water intake are signs of poisoning. Our study revealed that T-2 toxin is poisonous to the body, which is demonstrated by the reduction of BW, feed and water intake. Our finding was in line with a study reported that T-2 toxin decreased the BW (Zhang et al. 2022). We found that Ly curiously ameliorated T-2 toxin-induced BW loss, feed, and water consumption in mice. Villi, which resemble fingers and make up the gut mucosal structure, are crucial for fundamental health indicators as well as food absorption (Adam et al. 2024). Villi increase the small intestine's surface area, which is necessary for nutrient absorption (Hu et al. 2024). Since the small intestine is the first significant barrier that is faced following intake, it is especially susceptible to damage from mycotoxin (Liew and Mohd-Redzwan 2018; Ren et al. 2019). Previous studies reported that, a variety of harmful alterations, such as villus atrophy, crypt hyperplasia, epithelial cell degeneration, and changes in tight junction proteins, are revealed by histological analysis of the small intestine in mice exposed to mycotoxins. These changes all contribute to compromised intestinal barrier function and nutrient absorption (Li et al. 2025). Consistent with earlier research, we found that mice exposed to T-2 had increased CD and VW and decreased VH and VH: CD in the ileum, but Ly supplementation reversed these effects. Thus, using Ly supplements may partially reduce intestine damage caused by T-2 toxin.

The function of microbiota in gut health has drawn increased attention in recent years, and microbiota research has grown in importance (Álvarez-Cilleros et al. 2020). The composition of the gut microbiota is thought to be significantly influenced by the diet (Ye et al. 2019). Numerous studies demonstrated that dietary mycotoxin could potentially harm host organisms by efficiently modifying the intestinal flora (Liao et al. 2018). There is evidence that T-2 has a significant impact on bacterial populations (Li et al. 2024). The microbial communities of the four groups were clearly divided into distinct clusters for the intent of beta diversity analysis, indicating that the Ly and T-2 treatments had a considerable impact on the gut microbiota's beta diversities. Significant variations in the microbiota composition were found across all groups based on the examination of alpha and beta-diversity indices. Comparing the Ly, T-2, and T-2 + Ly groups to the Cont group revealed phylum-level changes in their gut microbial assemblages. When compared to the Cont group, the relative percentages of *Firmicutes* in the Ly, T-2, and T-2 + Ly groups were clearly higher, *Bacteroidota* was clearly lower, and *Actinobacteriota* was higher in the T-2 group than in the Cont, Ly, and T-2 + Ly groups. In contrast to our study, a study that examined the gut microbiota of rats exposed to T-2 toxin and selenium deficiency revealed that *Bacteroidetes* increased (11.11% vs. 14.94%) and *Firmicutes* were predominant but slightly reduced (81.39% vs. 77.06%) compared to controls. Generally, exposure to T-2 toxin reduces the abundance of *Actinobacteriota* (Wu et al. 2023). Some *Firmicutes* species have adaptations that allow them to survive and even thrive under such conditions, which may account for their increased relative abundance. This discrepancy may result from the fact that the relative abundance of some phylums can vary depending on the dosage and duration of T-2 toxin exposure as well as the particular experimental conditions (Wu et al. 2023). However, it was in agreement with the study that found T-2 toxin can interfere with the metabolism of bile acids, which can negatively impact the growth of *Bacteroidota*. Moreover, some *Bacteroidota* species may be directly inhibited from growing or killed by the harmful effects of T-2 toxin, which would reduce their relative abundance overall (An et al. 2025). Nevertheless, compared to T-2 mice, the T-2 + Ly group had higher prevalences of *Candidatus-Arthromitus*, *Staphylococcus*, and *Corynebacterium*. Notably, Ly supplementation partially altered the relative abundances of certain genera, though the biological significance of increased *Staphylococcus* and *Corynebacterium* requires further investigation. This was consistent with research showing that T-2 toxin can cause notable changes to the gut microbiome. *Staphylococcus*, for instance, greatly increased in abundance in the CUMS group, whereas Lactobacillus declined (Merchak et al. 2024; Zhao et al. 2024). T-2 toxin may cause a rise in the relative abundance of *Staphylococcus*, which could exacerbate inflammatory disorders (Zhao et al. 2024). Thus, the current investigation showed that the Ly therapies partially restored the gut microbiota dysbiosis caused by T-2.

According to the LEFSe analyses, the administration of Ly resulted in a decrease in the abundance of conditionally pathogenic bacteria, including *Bacilli, Staphylococcus*, and *Corynebacteriales*, indicating that the T-2 toxin treatment changed the microbial composition and upset the balance of the immune system. Our research supported earlier findings that different T-2 toxin treatment concentrations changed the microbial composition and upset the balance of the intestinal microbiota, as evidenced by younger mice's increased abundance of conditionally pathogenic bacteria like Escherichia *shigella* and *Anaerotruncus* (Kang et al. 2022)*.* Correlation network analysis revealed positive associations among several dominant phyla, including *Firmicutes, Proteobacteria, Actinobacteriota,* and *Bacteriodota*. These inter‑phylum positive correlations suggest that Ly supplementation may promote a more cooperative rather than competitive microbial ecosystem. However, because correlation does not imply causation, these associations should be interpreted as exploratory clues, for example, co‑occurring phyla might share similar ecological niches or metabolic dependencies rather than direct evidence of a mechanistic pathway. These findings showed a strong correlation between mice's gut microbiota imbalance and T-2 toxin exposure.

Tumor necrosis factor-α (TNF-α), and interleukin-1β (IL-1β) are examples of inflammatory cytokines that are essential to the inflammatory response (Miyashita et al. 2022; Huang et al. 2023). Through a variety of methods, exposure to T-2 toxin modulates the synthesis of pro-inflammatory cytokines. T-2 toxin has the ability to trigger the Janus kinase/signal transducer and activator of transcription (JAK/STAT) pathway, which increases the synthesis of pro-inflammatory cytokines, such TNF-α, IL-1β, and IL-18 (Huang et al. 2023). T-2 toxin-induced inflammation is facilitated via the PKA/CREB and NF-κB pathways, which also control the transcription of AKNA, a transcription factor linked to inflammation (Liu et al. 2017). Mice exposed to T-2 toxin experience cutaneous damage and skin inflammation, which raises reactive oxygen species and oxidative stress (Agrawal et al. 2012). DNA methylation affects T-2 toxin-induced liver damage, which is linked to the production of pro-inflammatory cytokines (Liu et al. 2019). All these were in agreement with our study which revealed that T-2 in mice significantly increased the concentration pro-inflammatory cytokines such as IL-1β, IL-2, IL-4, IFN-γ, IL-17, and TNF-α compared to Cont mice, while the Ly supplementation were significantly reduced their levels compared to T-2 mice. Microbiota and SCFAs possess anti-inflammatory properties, which can indirectly reduce oxidative stress. Inflammation is often associated with increased ROS production, and by mitigating oxidative stress, SCFAs can help to lower inflammation levels (Shin et al. 2023).

The broad definition of oxidative stress is a physiological stress caused by an imbalance between the antioxidant system's ability to scavenge reactive oxygen species (ROS) and the generation of ROS due to metal exposure (Adam et al. 2024). The consequences of this imbalance include changes in the redox state of cells, damage to macromolecules, and disruption of gene expression regulation (Sevcikova et al. 2011). The initial line of defense against oxidative stress is superoxide dismutase (SOD) activity, which transforms O_2_ and H + into less reactive H_2_O_2_ (Kim and Kang 2017). By converting H2O2 to water and oxygen, catalase (CAT) and GPH activities protect the majority of animals from oxidative stress. CAT also controls the generation of ROS within cells, which is linked to the control of cellular signaling (Smith et al. 2007). Furthermore, CAT is essential for preserving low H_2_O_2_ levels within the typical range, which promotes stress tolerance and cellular homeostasis (Adam et al. 2024). In order to determine the mechanism by which Ly supplementation enhances the antioxidant capacity, we examined the blood levels of a number of oxidative and antioxidant-related proteins. When compared to Cont, the activity of CAT, GSH, ATP, and SOD1 reduced in T-2 toxin mice, although the activity of ROS and MDA rose. However, when compared to T-2 mice, the addition of Ly returned their concentration to normal. Our results were consistent with other research showing that exposure to T-2 toxins increases the formation of ROS and reduces antioxidant defenses (Wang et al. 2023b). T-2 toxin may compromise the body's defenses against oxidative damage by inhibiting antioxidant enzymes such glutathione peroxidase (GSH-Px), CAT, and SOD (Sukalingam et al. 2018; You et al. 2021). The process promotes the formation of ROS by downregulating Nrf2, a master regulator of the antioxidant response (Chen et al. 2021). However, a reduction in SCFA production may result from gut microbial dysbiosis caused on by T-2 toxin (Huang et al. 2024). Decreased SCFA levels have the potential to accelerate systemic inflammation, raise oxidative stress, and damage the intestinal barrier. SCFA supplementation has demonstrated promise in reducing these side effects. For example, SCFAs enhanced lipid profiles and decreased inflammation and oxidative stress in a rat model of letrozole-induced polycystic ovarian syndrome (PCOS) (Acharya et al. 2024). Reduced SCFA synthesis and dysbiosis of the gut microbiota are closely related to systemic oxidative stress caused by T-2 toxin in mice. In order to reduce the harmful effects of T-2 toxin, Ly administration may be a viable strategy for modifying the gut microbiota, raising SCFA levels, and offering antioxidant protection.

Inflammatory processes and systemic autoimmune reactions have been associated with SCFAs, such as acetate, propionate, and butyrate, which are important metabolites generated by gut bacteria through the fermentation of dietary fiber. By preventing the release of pro-inflammatory chemicals, they also lessen intestinal inflammation by reducing inflammatory responses and reestablishing the intestinal barrier's functionality (Kim et al. 2013; Gill et al. 2018; Yao et al. 2024). When gut dysbiosis results in increased intestinal permeability, toxins from the gut can enter the bloodstream and cause oxidative stress and inflammation throughout the body (Mostafavi Abdolmaleky and Zhou 2024). By promoting a healthy gut flora, SCFAs can reduce oxidative stress and systemic inflammation while preserving the integrity of the gut barrier (Wang et al. 2021). Butyric, propionic, and acetic acids are the most common short-chain fatty acids in the GI tract, as shown by our mice investigations. SCFAs, particularly butyrate, can boost the synthesis of antioxidant enzymes like glutathione peroxidase (GSH-Px), catalase (CAT), and superoxide dismutase (SOD), which aid in reducing oxidative damage and neutralizing ROS (Zhu et al. 2024). T-2 toxin can cause dysbiosis of the gut microbiota, which is an imbalance in the beauty products and functionality of the gut microbial population (An et al. 2025). This dysbiosis may result in less SCFA being produced, which could affect the host's general health. In comparison to Cont mice, we found that T-2 markedly reduced the concentrations of hexanoic acid, butyric acid, isobutyric acid, isovaleric acid, acetic acid, propanoic acid, pentanoic acid, and heptanoic acid. Our finding was in line with previous study who reported that T-2 toxin exposure can altered the levels of various bacterial groups in the gut, which in turn affects the fermentation processes and SCFA synthesis (Kang et al. 2022; Huang et al. 2024; An et al. 2025). While the Ly administration significantly restored the SCFAs concentration compared to T-2 mice group.

The correlation analysis performed in this study provides a crucial integrative perspective, linking the modulation of gut microbiota and SCFAs by Ly to the amelioration of T-2 toxin-induced oxidative stress. The strong positive correlation between the pathobiont *Helicobacter* and ROS, coupled with its negative association with the antioxidant SOD1, suggests that this bacterium may be a key microbial contributor to the pro-oxidant environment following T-2 exposure. This is consistent with studies implicating *Helicobacter* species in promoting oxidative stress and inflammation in various gut pathologies [84, 85]. The restoration of a healthier microbial community by Ly, as seen by the reduction in *Helicobacter*, likely disrupts this pro-oxidative pathway.

Furthermore, the positive correlations between beneficial SCFAs (like butyric and acetic acid) and antioxidant enzymes (SOD1 and CAT), and their negative correlations with oxidative damage markers (MDA and ROS), strongly support the notion that SCFAs play a direct or indirect role in enhancing the host's antioxidant defense system. Butyrate, in particular, has been shown to activate the Nrf2 pathway, a master regulator of antioxidant gene expression [86]. Therefore, the ability of Ly to restore SCFA levels, as observed in our study, likely contributes to the attenuation of oxidative stress by bolstering these endogenous defense mechanisms. The positive association between *Bifidobacterium* and both antioxidants and SCFAs further underscores the interconnectedness of a healthy gut microbiome, its metabolic output, and systemic redox balance. In summary, these correlations suggest that the antioxidant efficacy of Ly against T-2 toxin is not a standalone effect but is mechanistically supported by its positive influence on the gut microbiota and its functional capacity to produce protective SCFAs.

The first limitation of this study was the small sample size because of the current resources, which may impact the generalizability of our results. Secondly, T-2 toxin significantly reduced feed and water intake, leading to body weight loss. Gut microbiota composition, SCFA production, oxidative stress, and inflammatory markers are all known to be influenced by nutritional status and caloric intake. Therefore, it remains unclear whether the protective effects of lycopene observed in this study are direct (e.g., via antioxidant or microbiota-modulating properties) or indirect (e.g., secondary to improved feed intake and overall health recovery). Thus, findings should be interpreted as hypothesis-generating rather than confirmatory. Therefore, to validate these findings and improve our comprehension of the phenomenon being studied, a larger number of samples and employing pair-feeding designs are required for future research.

## Conclusion

In conclusion, our research shows that Ly supplementation reduces T-2 toxin-induced oxidative stress and inflammation in mice, which is associated with modulation of the gut microbiota and increased fecal SCFA levels and improves ileal histomorphology. Compared with T-2 exposure alone, Ly supplementation improves body weight, feed and water intake, and ileal morphology (increased VH and VH:CD; decreased CD and VW). It also reduces several pro-inflammatory cytokines, restores antioxidant enzyme activities, and partially restores SCFA levels and gut microbiota composition. Future studies should investigate the translational potential of Ly supplementation for avoiding T-2 toxin-related health problems using larger sample sizes and include mechanistic validation to further clarify the underlying mechanisms.

## Materials and methods

### Experimental animals and materials

Twenty male BALB/c mice weighing 23.5 ± 0.32 g at 6 weeks of age were obtained from Yangzhou University in Yangzhou, China. The commercial diet was purchased from Jiangsu Xietong Pharmaceutical Bio-Engineering Co., Ltd. (Jiangsu, China) (Table 3). Ly was obtained from Shanghai Aladdin Bio-Chem Technology Co., LTD (Shanghai, China). T-2 was obtained from Qingdao Pribolab Biotech Co., Ltd (Qingdao, China), and the sunflower oil was obtained from market (Yangzhou, China).

**Table 3:** Product composition analysis guaranteed value for the basal diet (content per kg)

Items	Per kg
Moister	100 g
Grude protein	200 g
Grude fat	40 g
Coarse fiber	50 g
Coarse ash content	80 g
Calcium	10.18 g
Total phosphorus	12 g
Lysine	13.2 g
Methionine + cystine	7.8 g
VA	14,000 IU
Folic acid	6 mg
VE	120 IU
VD	1500 IU
Iron	120 mg
Manganese	75 mg
Zinc	30 mg
Selenium	0.2 mg

*VA* Vitamin A, *VE* Vitamin E, *VD* Vitamin D

### Experiment design

We randomly divided the mice into 4 groups (n = 5 per group) after 7-days adaptation before: Cont group given commercial diet as a basal diet, with administration of normal sunflower oil (0.3 mL). Ly group; we used sunflower oil to dissolve the Ly powder, and 100 µg/kg b.w. Ly was supplemented with 0.3 mL sunflower oil, the dose in each 0.3 mL was adjusted based on the mouse body weight. T-2 group; the same as Ly, we used sunflower oil to dissolve the T-2 powder, and supplemented at dose rate 200 µg/kg b.w. with 0.3 mL sunflower oil. These dosages were selected in light of earlier research showing that Ly has anti-oxidative and anti-inflammatory properties at comparable levels, that rodents can tolerate it, and that 200 µg/kg T-2 can cause repeatable toxin-related damage (Tang et al. 2007; Ahmed et al. 2022). Ly was administered 4 h after T-2 exposure (T-2 + Ly) group. This procedure was performed orally every 2 days for 35 days. Mice were housed in cages with a one-side self-feeder and a nipple water-feeder for free access to feed and water. Throughout the experiment, the mice were kept in a room with a 12-h light–dark cycle and a constant temperature of about 25 °C and 50% humidity. We reported weekly body weights, feed, and water consumption of the mice during the experiment. On the week 5, the mice were fasted overnight, and then in the morning anesthetized by using Sodium Pentobarbitone via intra peritoneal (i.p.) injection, then we collected blood and Fecal samples.

### Pro-inflammatory cytokines measurement

In the present study, ELISA kits from (Beijing Solarbio Science & Technology Co., Ltd.) were used to assess TNF-α using an assay kit (Cat:SEKM-0034), IL-1β using an assay kit (Cat:SEKM-0003), IL-2 using an assay kit (Cat:SEKM-0004), IL-4 using an assay kit (Cat:SEKM-0005), IFN-γ using an assay kit (Cat:SEKM- 0031), IL-6, using an assay kit (Cat:SEKM-0007), and IL-17 using an assay kit (Cat:SEKM-0018). We followed the manufacturer's recommendations for conducting the assays. In each well of a 96-well plate, we deposited the cytokines capture antibodies overnight. The next day, the plate's standard antigens were incubated with a second set of biotinylated antibodies prior to the addition of streptavidin. We recorded the color change from purple to yellow was measured at 450 nm, and their levels in each sample were expressed as (ng/L) for cytokine concentration.

### Oxidative and antioxidant indexes

We used commercial kits to identify the components and tested the following enzyme activity using the plasma: The plasma was analyzed using a PU 8720UV/VIS scanning spectrophotometer to measure the levels of MDA using an assay kit (Beyotime, S0131S, Shanghai, China), CAT using an assay kit (Beyotime, S0051, Shanghai, China), GSH using an assay kit (Beyotime, S0053, Shanghai, China), ATP using an assay kit (Beotime, S0026, Shanghai, China), and SOD1 activity using an assay kit with WST-8 (Beyotime, S0101S, Shanghai, China). In brief, the samples were treated with lysis buffer and then centrifuged. After that, the supernatants were mixed with TBA detection solution to measure the absorbance at 532 nm, which made it possible to calculate the MDA concentrations using the standard curve. Finally, data were normalized to tissue weight. Commercial kits obtained from Nanjing Jiancheng Bioengineering Institute (Nanjing, China) were used to measure concentrations. We used the OxySelect Assay Kit (Cell Biolabs, STA-347, San Diego, CA, USA) to examine ROS. ROS levels in plasma were determined by detecting the highly fluorescent Dichlorodihydrofluorescein (DCF) from the oxidation of 2,7-dichlorodihydrofluorescein (DCFH) by ROS at 480 excitation and 530 nm emissions.

### Intestinal morphology

The segment of small intestinal samples (ileum) was fixed in paraformaldehyde solutions for 48 h. It was then cleaned, removed, and subjected to a dehydration and rehydration process before being embedded in paraffin. After cutting the tissues to a 5 µm thickness, hematoxylin–eosin staining was applied. Six well-oriented villus–crypt units were selected per sample, and measurements were performed in a blinded manner. Images were captured with an Olympus microscope (U-TV0.63XC, Tokyo, Japan) and sections were examined using a 10 × objective lens. Villus height (VH) and width (VW) distances from the villus tip to the crypt, crypt depth (CD) distances from the villus base to the submucosa, and the ratio of villus height to crypt depth (VH/CD) were among the intestinal morphological parameters that were measured using the Infinity Analyze software (Version7, Lumenera Corporation, Ottawa, ON, Canada).

### 16S rRNA sequence analysis

The mice were kept in sterile cages in a barrier environment and allowed to defecate naturally. After collecting the voided feces with sterile forceps, the samples were labeled appropriately and put into 1.5 mL sterile EP tubes. The samples were immediately preserved at −80 °C after being collected. At least six fecal pellets were collected from each mouse in order to guarantee a sufficient sample volume for examination. The following procedure was used to perform 16S rRNA sequencing. The QIAamp DNA Stool Mini Kit (Qiagen, Düsseldorf, Germany) was used to extract DNA from fecal samples. Agarose gel electrophoresis and a NanoDrop 2000 (Thermo Fisher Scientific, Waltham, MA, USA) were used to evaluate the integrity and purity of the extracted DNA. The 16S rRNA V3-V4 region was then amplified by PCR using primers 515 F (5′′-GTGCCAGCMGCCGCGGTAA 3′′) and 806R (5′′ GGACTACHVGGGTWTCTAAT-3′′). After that, PCR products from each group were purified and quantified. The TruSe qTMDNASamplePreparation Kit was used to create the libraries, and the HiSeq PE250 platform (Illumina, San Diego, CA, USA) was used for sequencing. Fastp software (version 0.19.6, logon date: 2023–11-05) was used for quality assurance. FLASH software (version 1.2.7) was used for sequence assembly (Magoč, T., and Salzberg, S. L. 2011), and the amplicon sequence variants (ASVs) were generated using the DADA2 (v2.8.1) pipeline. Based on ASV data, species were annotated and then examined for species differentiation, community diversity, and composition (phyloseq, v1.46.0).

### Short-Chain Fatty Acid (SCFAs) profile in feces

We used Ultra-High Performance Liquid Chromatography-Tandem Mass Spectrometry (UPLC-MS/MS) to quantify and analyze the relative amounts of SCFAs including hexanoic acid, butyric acid, isobutyric acid, isovaleric acid, acetic acid, propionic acid, pentanoic acid, and heptanoic acid, in the feces of Cont mice and that following T-2 and/or Ly administration. In brief, approximately fifty milligrams of freshly collected feces were mixed with 0.5 ml of water, and the mixture was ultrasonically homogenized for 10 min. After adding 0.5 ml of internal standard solution (2-ethylbutyric acid, 5 μg/mL) to facilitate extraction, the mixture was vortexed for half an hour. The sample was then centrifuged for 5 min at 4 °C and 12,000 revolutions per minute (rpm). Then, 50 µl of the supernatant were combined with 50 µl of an isotopically tagged internal standard (5 µg per milliliter). Additionally, 50 µl of 3-nitrophenylhydrazine (3-NPH) solution (250 mM, prepared in a 50% methanol/water solution) and 50 µl of 1-ethyl-3-(3-dimethylaminopropyl) carbodiimide (EDC) solution (150 mM, prepared in a 75% methanol/water solution containing 7.5% pyridine, resulting in a ratio of methanol: water: pyridine = 69.375:23.125:7.5). The mixture was then placed on a shaker and allowed to react at 30 °C for 30 min. Next, 50 µl of a butylated hydroxytoluene (BHT) methanol solution (2 mg per milliliter) and 250 µl of a 75% methanol/water solution were added. A low-temperature centrifuge was then used to spin the centrifuge tube for five minutes at 12,000 rpm and 4 °C. After that, 100 µl of the supernatant were taken out and placed in an injection vial for subsequent mass spectrometry analysis. A Waters UPLC BEH C8 chromatographic column (100 mm ×2.5 mm ×1.7 µm) was utilized. For quantification, calibration curves were constructed using authentic standards at concentrations ranging from 0.1 to 1000 µM with 7–9 points, and all analyte concentrations were normalized to wet fecal weight and expressed as μmol/g wet feces. The method was validated with recovery rates ranging from 83% to 108%, precision (relative standard deviation) of less than 10%, and limits of detection (LOD) and quantification (LOQ) of 0.4–3.8 and 1.9–8.3 µM, respectively, for each SCFA. A blank sample and a quality control sample were included in each batch to ensure method reliability.

### Statistical analysis

QIIME2 (version 2023.5) was used to analyze the sequencing data in order to determine whether or not the gut microbial community's diversity and structure differed across the different treatment groups (Srivastava et al. 2024). Furthermore, FASTP (version 0.18) was used to process the raw data in the following steps: readings with at least 10% of N bases removed; readings with at least 50% of bases and a Phred score of less than or equal to 20 removed; and readings with adapters discarded (Chen et al. 2018). Primer sequences from the cleaned reads were trimmed using the UPARSE program, and a 97% similarity threshold was applied. The QIIME tool was used to pick and annotate representative sequences for each OTU before beginning the 16S rRNA investigation. Taxonomic classification was performed using the SILVA (version 23) database with the RDP classifier (McDonald et al. 2024).

Diversity indices (Chao1, Shannon, Simpson, Good’s coverage, and Pielou’s evenness) were calculated using the R software (https://mothur.org/wiki/calculators/), to analyze whether the species diversity differences between groups were significant. Together with the rank-abundance curves, the diversity index dilution curves were plotted using the ggplot2 tool (Wickham 2011). An analysis of differences in the alpha index between two and many groups was conducted using the Vegan program. Beta-diversity analyses (PCoA, NMDS, and UPGMA) were performed using Bray–Curtis distance (Dixon 2003). In accordance with the Encyclopaedia of Genes and Genomes' recommendations, linear discriminant analysis (LDA) of effect size (LEfSe) was used to determine differentiating taxa and projected functions between the treatment groups (Khleborodova et al. 2024).

IBM SPSS Statistics version 26 was utilized to do statistical analysis. The groups' differences in body weight feed consumption and water intake were assessed using a one-way analysis of variance (ANOVA). To pinpoint the exact differences between the treatment groups, multiple comparisons were subjected to Tukey's post hoc test. The following p-values were provided for each significant comparison based on the results of the statistical study. *P* < 0.05 was considered statistically significant, and false discovery rate (FDR) correction was applied where appropriate.

To investigate the potential relationships between oxidative stress, gut microbiota, and fecal SCFAs, Spearman’s rank correlation analysis was performed; however, these associations do not imply causality. The mean values for each oxidative stress marker (ROS, MDA, CAT, GSH, ATP and SOD1), the relative abundance of key microbial taxa, and the concentration of each SCFA from all individual samples across the four experimental groups were used. Correlation coefficients (r) and p-values were calculated. A p-value of less than 0.05 was considered statistically significant for these correlations.

## Data Availability

The data presented in this study are available from the corresponding author upon request.

## Abbreviations

16S rRNA: 16S Ribosomal RNA
ASV: Amplicon Sequence Variant
ATP: Adenosine Triphosphate
BHT: Butylated Hydroxytoluene
CAT: Catalase
CD: Crypt Depth
DCF: Dichlorofluorescein
DCFH: Dichlorodihydrofluorescein
EDC – 1: Ethyl-3-(3-dimethylaminopropyl) carbodiimide
GIT: Gastrointestinal Tract (implied)
GSH: Glutathione
IACUC: Institutional Animal Care and Utilization Committee
IFN-γ: Interferon gamma
IL-1β: Interleukin-1 beta
IL-17: Interleukin-17
JAK/STAT: Janus kinase/signal transducer and activator of transcription
KEGG: Kyoto Encyclopedia of Genes and Genomes
LDA: Linear Discriminant Analysis
LEfSe: Linear Discriminant Analysis Effect Size
Ly: Lycopene
MDA: Malondialdehyde
MRPP: Multi-Response Permutation Procedure
NMDS: Non-Metric Multidimensional Scaling
OTU: Operational Taxonomic Unit
PCoA: Principal Coordinates Analysis
PCA: Principal Component Analysis
PKA/CREB: Protein Kinase A/ cAMP response element-binding protein
PCR: Polymerase Chain Reaction
ROS: Reactive Oxygen Species
SOD1: Superoxide Dismutase1
T-2: Trichothecene 2
TNF-α: Tumor Necrosis Factor-alpha
UPGMA: Unweighted Pair Group Method with Arithmetic Mean
VH: Villus Height
VW: Villus Width
